# Estimation of the global burden of tungiasis in endemic countries using a disability adjusted life-years modelling approach

**DOI:** 10.1186/s44263-026-00318-2

**Published:** 2026-08-31

**Authors:** Alistair Antonopoulos, Lynne Elson, Jürgen Krücken, Marlene Thielecke, Francis Mutebi, Richard B. Yapi, Ulrike Fillinger, Hermann Feldmeier, Ruth Nyangacha, Nono-Raymond Swar Kuispond, Eddy Kakiese Laken, Renata Santiago Alberto Carlos, Johannes Charlier, Georg von Samson-Himmelstjerna

**Affiliations:** 1 Kreavet, Kruibeke, Belgium; 2 Kenya Medical Research Institute (KEMRI)-Wellcome Trust Research Programme, Kilifi, Kenya; 3 Nuffield Department of Medicine, University of Oxford, Oxford, United Kingdom; 4 Institute for Parasitology and Tropical Veterinary Medicine, Freie Universität Berlin, Berlin, Germany; 5 Centre for Resistance Research, Freie Universität Berlin, Berlin, Germany; 6 Institute of International Health, Charité Center for Global Health, Charité University Medicine Berlin, Berlin, Germany; 7 College of Veterinary Medicine, Animal Resources and Biosecurity, Makerere University, Kampala, Uganda; 8 Centre Suisse de Recherches Scientifiques en Côte d’Ivoire, Abidjan, Côte d’Ivoire; 9 Centre d’Entomologie Médicale et Vétérinaire, Université Alassane Ouattara, Bouaké, Côte d’Ivoire; 10 Institute of Human Development, Aga Khan University, Nairobi, Kenya; 11 Institute of Microbiology, Infectious Diseases and Immunology, Charité - University Medicine, Berlin, Germany; 12 Center for Traditional Medicine and Drug Research, Kenya Medical Research Institute (KEMRI), Nairobi, Kenya; 13 National Institute of Biomedical Research, Kinshasa, Democratic Republic of the Congo; 14 Pathology Department, University of Kinshasa, BP127 KIN XI Kinshasa, Democratic Republic of the Congo; 15 Programme National de Lutte Contre le Paludisme (PNLP), Kinshasa, Democratic Republic of the Congo; 16 Universite de la Paix, Commune de la N’Sele, Democratic Republic of the Congo; 17 Departamento de Ciências Agrárias e Ambientais, Universidade Estadual de Santa Cruz (UESC), Ilhéus, Brazil

**Keywords:** Tungiasis, DALYs, Years lived with disability (YLDs), Burden, Endemic countries

## Abstract

**Background:**

Tungiasis is a neglected tropical zoonotic skin disease which is endemic to Sub-Saharan Africa and Latin America, caused by sand fleas of the genus *Tunga*, which burrow into the skin of affected humans and animals, most commonly when the feet come in contact with infested soil. Sand flea infection leads to inflammation, severe itching, and pain, with the potential for secondary bacterial or viral infections, gangrene, and disfigurement. Tungiasis disproportionately affects poor rural communities, and individuals living in unhygienic settlements. To date, the existing burden of tungiasis has never been estimated, despite being included in the World Health Organisation Neglected Tropical Diseases roadmap since 2021.

**Methods:**

We applied a prevalence-based Disability Adjusted Life Year (DALY) approach focusing on Years Lived with Disability (YLD) to model the burden of human tungiasis in endemic countries. Prevalence range estimates were derived using a Bayesian hierarchical Beta-Binomial model, with probability distributions applied to represent uncertainty in estimates based on highly heterogeneous local reports, capture local and regional variation, and reflect fluctuations in symptom severity among affected individuals. A discrete time point model was implemented over a 52-week period to estimate annual DALYs lost per country included in the analysis. Monte Carlo simulation with 1000 iterations was used to generate mean DALY estimates.

**Results:**

Globally, we estimate that up to 379,645 (95% UI 361,792–398,309) DALYs are lost annually, with the bulk of the burden in Sub-Saharan Africa. When comorbidities are included, this increases to 575,298 (95% UI 548,948–602,597). When adjusted for population size, we estimate that 24.2 DALYs (36.4 with comorbidities included) are lost annually per 100,000 people. In terms of DALYs lost, the disease burden is disproportionately concentrated in Sub-Saharan Africa, and Brazil.

**Conclusions:**

Our estimates suggest tungiasis exerts a substantial burden of disease in endemic countries, comparable in absolute terms to that of soil-transmitted helminths and echinococcosis, and greater than that of toxocariasis, fascioliasis. Furthermore, we observe marked regional concentration of disease burden within Sub-Saharan Africa, with the disease burden in this region driven by large at-risk rural, and urban slum-dwelling, populations living within the ecological zone of transmission.

## Background

Tungiasis is a neglected tropical disease (NTD) endemic to Sub-Saharan Africa, Central and South America, and certain parts of the Caribbean [1]. It is caused by adult female sand fleas of the genus *Tunga* (*Tunga penetrans* and *Tunga trimamillata*) which burrow into the skin, most often on the feet. The female, once embedded into the skin remains in contact with the environment via its last abdominal segment through a 250–500 μm opening in the skin, which allows it to breathe, excrete, copulate while fixed in the skin, and shed eggs into the environment. This further increases the risk of secondary bacterial or viral infections due to breaks in the skin. Inflammation occurs due to the rapid growth of the embedded sand fleas, leading to pressure on the surrounding tissues [2]. Fertilised females can expand in size up to 2000-fold in around a week due to growth of eggs in the abdomen, leading to pain and inflammation, which can cause difficulty of walking, in addition to insomnia and issues of concentration [3]. Further inflammation may be related to endosymbiotic bacteria, *Wolbachia* [4], particularly due to the release of *Wolbachia*-derived lipopolysaccharides into the surrounding tissue upon the death of the parasite [1]. This inflammatory pathophysiology can lead to suppuration, abscesses, ulcers, lymphangitis, necrosis, and potentially gangrene and in chronic cases, disfigurement, disability, and difficulty in walking [1, 2]. This can be exacerbated by unhygienic manual extraction, often with tools such as safety pins, needles, scissors, knives, or sharp pointed pieces of wood [5]. These practices are painful, and can often lead to secondary infections such as tetanus, HIV, hepatitis B and C [6]. Tungiasis has also been implicated in contributing to school absenteeism, poor school performance and lower neurocognitive function [3, 7–9]. Tungiasis also has a strong impact on the self-perception of affected individuals leading to a decreased quality of life [3, 10].

*Tunga penetrans* and *T. trimamillata* are zoonotic parasites, and animal tungiasis often occurs alongside human infections. This is relevant both for the potential impact on livestock productivity, such as in swine or small ruminants [11], and because infections in animals can serve as reservoirs for human disease [12–14]. Tungiasis is highly prevalent in rural areas of Sub-Saharan Africa, where 668 million people are estimated to live in risk areas, which are ecologically conducive for transmission [15, 16]. The disease disproportionately affects resource-poor rural communities. Residents of urban and peri-urban informal settlements are also predisposed to tungiasis [17]. Accordingly, tungiasis prevalence is strongly associated with socioeconomic factors [8, 9, 18], particularly poor hygiene and sanitation [19–21]. Reported prevalence varies widely depending on study design and setting. National-level data from Kenya indicate a prevalence of 1.35% (1.15–1.59%) [22], whereas a systematic review and meta-analysis of studies conducted in targeted high at-risk communities across Sub-Saharan Africa between 1980 – 2019 reported a pooled prevalence of 33.4% (27.6–39.8%) [23]. Prevalence appears to be even higher among school-aged children, with a recent meta-analysis estimating a pooled prevalence of 37.9% (31.0 – 44.8%) in affected communities [17]. Exceptionally high prevalences have been documented in villages in the Karamoja region of northeastern Uganda, where prevalence reached 84.6% in some villages [24]. Particularly in East Africa, comorbidity with podoconiosis has also been reported, with between 10-20% of individuals in certain communities affected by both conditions [25].

In Latin America, approximately 450 million people are estimated to live in at-risk areas [26]. A systematic review and meta-analysis of Brazilian studies reported prevalence ranging from 1.6% to 54.8%, predominantly among children aged 5 – 9 [27]. Across the Americas prevalence estimates range from 1.0 to 82.6%, reflecting substantial environmental and socioeconomic heterogeneity [28]. Notably, most available data is derived from studies conducted in purposely selected high-risk communities, with only one study to date estimating national prevalence using semi-random sampling of primary schools [22]. Furthermore, tungiasis prevalence varies temporally with peak transmission during dry seasons [29]. Substantial changes have been described in short time periods during a single dry season [14].

The disability adjusted life year (DALY) is an indicator of the burden of disease which was first used as part of the ongoing global burden of disease (GBD) study in 1992 [30]. The DALYs are calculated based on the sum of years of life lost (YLL), and years lived with disability (YLD) [30]. The DALY based approach has been instrumental in showing the true burden of neglected tropical diseases (NTDs), mental health disorders, chronic pain and disability. Burden of disease estimation using the DALY approach has also been identified as an important contributor to the construction of research agendas [31, 32]. The burden of many diseases were previously underestimated because they cause little mortality. To inform tailored control strategies of parasitic diseases with substantial morbidity, DALYs have been used extensively to estimate disease burden for many infections, including soil-transmitted helminths (STHs) [33], schistosomiasis [34], echinococcosis [35], fascioliasis [36], toxocariasis [37], and ectoparasites such as scabies [38]. However, tungiasis has not yet been considered in burden of disease studies, despite its inclusion in the WHO NTD roadmap since 2021 [39], and an estimated population of approximately 1.1 billion living in areas ecologically conducive to transmission [15, 16, 26]. In this study, we therefore, aim to provide a preliminary estimate of the global burden of tungiasis in endemic countries using a DALY-based years lived with disability (YLD) approach.

## Methods

### Model overview

**Fig. 1:**
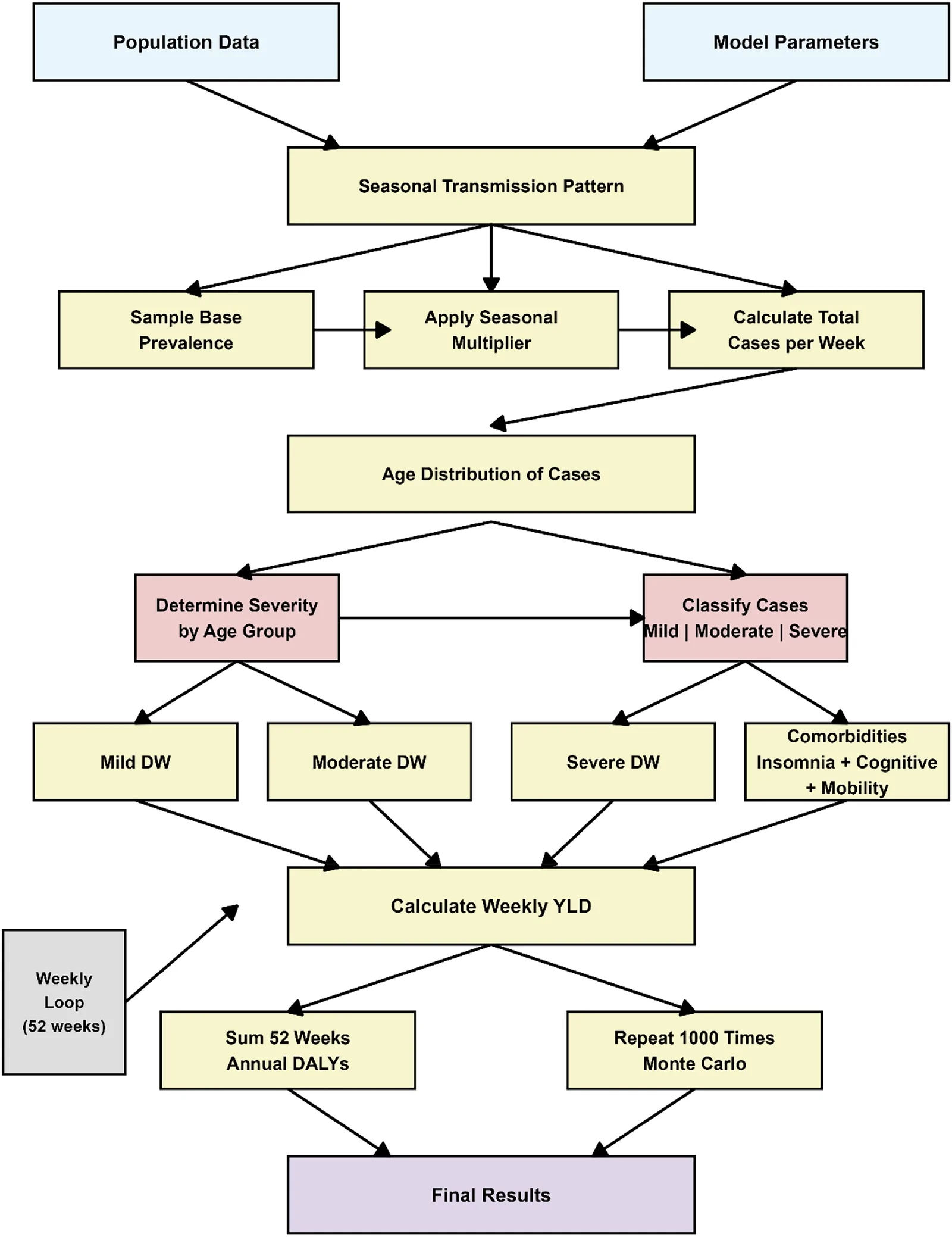
Flow diagram summarising the model structure for the estimation of the global burden of tungiasis. DW – disability weight; YLD – years lived with disability; DALY – disability adjusted life years. Weekly YLD is based on weekly resampling DWs

We used a prevalence-based DALY model to estimate the annual global burden of tungiasis. Because tungiasis is primarily a non-fatal but highly morbid condition, the model focused on years lived with disability (YLDs). Associated complications can be fatal; however, we have not considered these in the current model due to the difficulties in parameterising this aspect and directly attributing deaths to comorbidities. To account for uncertainty and variability, ranges are assigned and the model samples across the distribution (program evaluation and review technique (PERT) or Uniform, depending on the context) for key input parameters such as prevalence within the at-risk population, and disability weights (DW) to model stochasticity. This reflects the variable nature of the symptoms experienced by individuals suffering from the disease at a given time, and the propensity for undulating infection and re-infection following manual removal or treatment of the sand fleas. The DALY model is run for a time period of 52 simulated weeks to yield an annual estimated disease burden. Time points for data collection are each week of the 52-week year, and at the end of each iteration, the total DALYs were summed for that year. The simulation ran for 1000 iterations in total, with values averaged (mean; median: Supplementary Material 1). This allowed for the estimation of an annual rate of DALYs lost. The final calculation of the DALYs was expressed as the mean DALYs across all simulations, and the 95% uncertainty intervals based on the data from all iterations. The prevalence in any one week of the 52-week simulation was estimated by sampling across a PERT distribution of this range. This was to reflect the varying intensity of the infection in the population at large, and to reflect the fact that infection intensity may vary as fleas are removed and feet become re-infected. Additionally, the variation in the severity of infection is also modelled by probability distribution sampling across the DW ranges. A summary of the model framework is shown in Fig. 1. Although tungiasis is zoonotic, we have not modelled this aspect, as the current model focuses on a prevalence-based DALY approach, and we do not consider transmission from animals or the environment as a component of the model, rather, focusing on potential prevalence ranges.

### DALY calculations

In this study, we apply a modified prevalence-based DALY framework that considers only years lived with disability (YLD) [30]. We adopted this approach because potential indirect mortality from secondary infections was not included, owing to both the complexity of reliably estimating such outcomes and the lack of data to adequately parameterise them. Accordingly, the analysis is restricted to morbidity outcomes that can be directly attributed to tungiasis infection, such as, but not limited to, insomnia resulting from pruritus (Table 1). Under this framework, DALYs lost are calculated as follows:

$$\begin{array}{lll}{\rm{DALY}}(c,s,a,t)&=&{\rm{YLD}}(c,s,a,t)\\&&{\rm{for\; given\; cause\;c}},\\&&{\rm{age\;a}},{\rm{sex}}\;{\mathrm{s}}\:{\rm{and\; year}}\;{\mathrm{t}}\end{array}$$

Where:

YLD(c,s,a,t) = P(c,s,a,t) x DW(c,s,a)^*^ x L(c,s,a,t)

P(c,s,a,t) = number of prevalent cases for cause c, age a and sex s

DW(c,s,a) = disability weight for cause c, age a and sex s

L(c,s,a,t) = average duration of the case until remission in the reference year

^*^ For comorbidities, the GBD multiplicative rule 1 − Π(1 − DWᵢ) is applied

**Table 1:** Model input parameters and distributions

Parameter Category	Parameter	Distribution Type	Values	Unit
**Prevalence**				
Regional Prevalence (*p*_*c*_*)*	Tungiasis prevalence	PERT	Region specific (ESA, WCA, SA, CAC, Supplementary Material 4)	% of at-risk population
**Seasonal Variation**				
Peak month	Transmission peak	Fixed	Month 3	Calendar month
Trough month	Transmission trough	Fixed	Month 9	Calendar month
Peak multiplier	Maximum transmission	Fixed	1.50 (+50%)	Relative to baseline
Trough multiplier	Minimum transmission	Fixed	0.5 (-50%)	Relative to baseline
**Age Distribution**				
Ages 0-4 years	Proportion of cases	Fixed	26%	Proportion of total cases
Ages 5-14 years	Proportion of cases	Fixed	34%	Proportion of total cases
Ages 15-59 years	Proportion of cases	Fixed	17%	Proportion of total cases
Ages 60+ years	Proportion of cases	Fixed	23%	Proportion of total cases
**Severity Distribution**				
Ages 0-4	Moderate-severe rate	Uniform	Min: 33%, Max: 55%	Proportion of age group cases
Ages 5-14	Moderate-severe rate	Uniform	Min: 33%, Max: 55%	Proportion of age group cases
Ages 15-59	Moderate-severe rate	Fixed	30%	Proportion of age group cases
Ages 60+	Moderate-severe rate	Uniform	Min: 36%, Max: 60%	Proportion of age group cases
All ages	Severe complications	Fixed	20% of moderate-severe	Proportion of mod-severe cases
**Disability Weights**				
Mild tungiasis	Disability weight	PERT	Min: 0.005, Mode: 0.011, Max: 0.021	Unitless (0-1)
Moderate tungiasis	Disability weight	PERT	Min: 0.015, Mode: 0.027, Max: 0.042	Unitless (0-1)
Severe tungiasis	Disability weight	PERT	Min: 0.125, Mode: 0.188, Max: 0.267	Unitless (0-1)
**Comorbidities**				
Insomnia (severe cases)	Disability weight range	Correlated	Min: 0.148, Max: 0.219	Unitless (0-1)
Cognitive impairment	Disability weight range	Correlated	Min: 0.018, Max: 0.050	Unitless (0-1)
Insomnia frequency	Days per week	Uniform	Min: 2, Max: 6	Days/week
Impaired mobility	Disability weight range	Correlated	Min: 0.054, Max: 0.11	Unitless (0-1)
**Population Parameters**				
At-risk definition	Rural populations (*R*_*c*_) + urban slum populations (*S*_*c*_)	Variable	Country-specific	Population count
Age stratification	Population groups	Fixed	0-4, 5-14, 15-59, 60+	Years

In the present model, the duration parameter (L), is fixed at 1, as the unit of simulation is one week within a 52-week period. At the population level, at any given week, we assume that the prevailing severity of disease manifestation at any given time point persists for one week. This structure allows the model to capture dynamic changes in infection severity across the population over time, reflecting cycles of symptom fluctuations due to flea removal, with periods of potentially low or absent symptoms or potential worsening of symptoms, and subsequent reinfection throughout the year. While this approach necessarily simplifies the heterogeneous disease trajectories experienced by individuals, it was adopted to enable estimation of disease burden at the global scale and should be interpreted in that context.

### General model structure and assumptions

To model the spectrum of symptoms experienced by patients with tungiasis, we define three distinct disease manifestations, with partially overlapping symptomatic DW ranges. These are modelled by sampling across a distribution, with variation occurring each week of the simulation. Each of these manifestations represents a dynamic range that may fluctuate over the course of several weeks or months. However, we recognise that, as with many diseases, these divisions are somewhat artificial but are a necessity for the purposes of quantification. On the other hand, we believe that these proposed DWs represent a best current approximation of the symptomatic burden of tungiasis.

### Disability weights

All DWs used in this study are derived from the Global Burden of Disease study (2009–2019) [40]. A full breakdown of all DWs for the associated global burden of disease study can be found in Supplementary Material 2. We assign DWs for tungiasis based on existing DWs for conditions with similar symptoms.

Mild tungiasis is defined as a dynamic range of relatively low level of symptoms attributable to infection that does not substantially affect the individual’s daily functioning, including sleep, mobility, or attendance at work or school and corresponds to individuals having less than five embedded fleas [41]. For this category, we have used the DW for “mild onchocerciasis”, disfigurement level 1: *slight physical deformity that others notice, causes some worry and discomfort*. DW = 0.011 (0.005–0.021) [38]. Although we recognise that mild tungiasis may lead to some sleep disturbance, this was judged to be below the threshold for the DW for insomnia and is thus covered within the DW for disfigurement level 1.

Moderate to severe tungiasis (without complications) is defined as a dynamic range of moderate to severe level of symptoms attributable to infection that progressively affects daily functioning, sleep, mobility, and attendance at work or school and corresponds to individuals with 6 to 30 embedded fleas. For this category, we have used the DW for scabies, disfigurement level 1 with itch/pain: *slight visible physical deformity that is sometimes sore or itchy. Others notice the deformity, which causes some worry and discomfort*. DW = 0.027 (0.015 – 0.042) [38].

Severe tungiasis is defined as a dynamic range of the highest levels of symptoms attributable to infection, which substantially impacts daily functioning, sleep, mobility, and attendance at work or school, and corresponds to individuals with more than 30 embedded fleas. For this category, we have used the DW for “severe skin disease due to onchocerciasis”, disfigurement level 2 with itch/pain: *visible physical deformity that is sore and itchy. Others may stare and comment, causing worry. The individual has trouble sleeping and concentrating*. DW = 0.188 (0.125 – 0.267) [38].

### Comorbidity disability weights

Tungiasis can lead to a range of comorbidities due to the symptoms of the disease that directly affect an individual’s day-to-day life beyond the primary manifestations. Thus, in case of severe tungiasis, we also include an additional DW encompassing the severe effects of tiredness and pain resulting from intense itching, and inability to sleep. For this, we adopted the DW for “post-dengue chronic fatigue syndrome”, as it further captures the psychological and emotional consequences of chronic and severe manifestations of the infection: *is always tired and easily upset; the person feels pain all over the body and is depressed*. DW = 0.219 (0.148–0.308) [38].

For individuals between the ages of 5–15, we further assigned a DW serving as a proxy for missed school attendance and difficulty concentrating due to the effects of the infection. For this we used the DW for “mild cognitive impairment” previously applied to model a similar situation in toxocariasis (DW = 0.031 [0.018 – 0.05]) [38]. This is also in line with previous work demonstrating impacts on neurocognitive function and school performance associated with tungiasis infection [8, 9].

Finally, for the most severe cases, as impaired mobility was not considered in the assigned DW, we included an additional DW for impaired mobility due to Guinea worm disease: *“has moderate pain in the leg, which makes the person limp and causes some difficulty walking, standing, lifting and carrying heavy things, getting up and down, and sleeping”*. DW = 0.079 (0.054–0.11) [38].

Duration ranges were introduced for severe cases, reflecting the likelihood that insomnia varies between individuals, and that affected individuals may be able to sleep on some nights during the week. These ranges depend on infection severity, thus, as the severity increases, the likelihood of longer durations of insomnia also increases, ranging from 1–7 nights per week of being unable to sleep due to pain or itching caused by the infection. The range of insomnia and associated psychological consequences is explicitly tied to the value within the severe tungiasis DW range. For example, if an individual’s DW is 0.125 the associated DW for additional disease consequences is 0.148 (i.e. both values occupy the equivalent position within their respective ranges). We applied the DW for insomnia only to individuals experiencing severe tungiasis. This represents an assumption that may not fully reflect reality. However, in the absence of specific data to quantify this effect, we adopted a conservative approach to reduce the risk of overestimation. We acknowledge that insomnia of varying severity may be present in many individuals with tungiasis, irrespective of the disease severity. However, in this analysis the DW was interpreted as representing only the most severe manifestations of insomnia, which lead to significant consequences for the affected individual.

### Seasonality

Seasonality of transmission has previously been identified in an empirical study from Brazil [29]. In this study, tungiasis prevalence in a community was reported to be 54.4% in September, at the peak of the dry season, and 28.8% seen in June, at the end of the wet season [29]. We, therefore, chose to model a seasonal component in this study. This was achieved by applying a sigmoidal pattern to a multiplication factor that modifies the prevalence range by week of the year, with the “dry season” peak at month three, and the “wet season” peak at month 9, as follows:

$$\begin{array}{lll}{\rm{Prevalence}}\,* \,{\rm{seasonal}}\,{\rm{multiplier}}&=&{\rm{seasonal}}\,\\&&{\rm{prevalence}}\\&&\,[{\rm{week}}\,{\rm{x}}]\end{array}$$

However, this seasonal adjustment was applied uniformly across all countries. We did not account for regional variations in the duration or intensity of dry seasons, nor for the presence of multiple wet and dry seasons, as occur in many tropical regions. Instead, we assumed an average seasonal variation.

### Estimation of the number of infections

To generate background prevalence estimates, we selected prevalence estimates from two reviews published for Sub-Saharan Africa [42], and South America [43], followed by an additional literature search for additional countries not included in the available systematic reviews (Supplementary Material 3; Table S1; Figure S1; Figure S2). To generate regional prevalence estimates to be applied at the national level across the at-risk population from heterogeneous primary study data, we employed a Bayesian hierarchical Beta-Binomial model. This approach treats the observed case counts at each study site as Binomial draws from a site-specific true prevalence, which is itself drawn from a regional Beta distribution, allowing partial pooling of information across sites within each region. Partial pooling allows data-sparse countries to borrow statistical strength from regional data while still permitting country-level deviation from the regional mean, deemed a necessity here where surveillance data are geographically uneven. The model was specified with two hierarchical levels: a regional level, capturing the distribution of true prevalence across West and Central Africa, East and Southern Africa, South America, and Central America and the Caribbean separately, and a site level, capturing within-region heterogeneity driven by setting type, and study design. A national-level prevalence anchor was incorporated using the available Kenyan national survey estimate [22], from which a setting correction factor was derived to translate focal high-transmission estimates to national-level equivalents for countries lacking national survey data. Posterior distributions were estimated using Markov Chain Monte Carlo (MCMC) sampling via Stan, implemented in R (R version 4.5.1 (2025-06-13 ucrt) through the brms (2.23.0) package with fixed random seed 123. Weakly informative Beta (1,1) priors were specified at the regional level, allowing the data to determine the regional distribution. Posterior predictive checks were conducted to assess model fit, and convergence was confirmed using the Gelman-Rubin diagnostic (R-hat < 1.01) and effective sample size (ESS) assessment. The Beta-Binomial hierarchical structure was chosen over fixed-effects meta-analysis as tungiasis prevalence studies exhibit substantial between-study heterogeneity driven by setting type, transmission intensity, and population vulnerability.

#### Observation level

For each study site i, with ${n}_{i}$ individuals examined and ${k}_{i}$ positive cases:

$${k}_{i}\sim {\rm{Binomial}}\,({n}_{i},{p}_{i})$$

where ${p}_{i}$ is the true underlying prevalence at site i.

#### Site-level model

The site-level prevalence is modelled on the logit scale as:

$${\rm{logit}}\,({p}_{i})={\mu }_{r[i]}+{u}_{c[i]}$$

where ${\mu }_{r[i]}$ is the fixed effect for the subregion r to which site i belongs (ESA, WCA, SA, or CAC), and ${u}_{c[i]}$ is the country-level random effect for country c.

#### Random effects

$${u}_{c}{\mathscr{\sim }}{\mathscr{N}}(0,{\sigma }_{c}^{2})$$

where ${\sigma }_{c}$ is the between-country standard deviation, estimated from the data.

#### Overdispersion

Because tungiasis prevalence studies exhibit substantial between-site heterogeneity beyond what the Binomial likelihood can accommodate, the observation model is extended to a Beta-Binomial:

$${k}_{i}\sim {\rm{Beta}}-{\rm{Binomial}}\,({n}_{i},{p}_{i},\phi )$$

where ϕ>0 is a precision parameter controlling overdispersion. As $\phi \to \infty$ the Beta-Binomial converges to the Binomial.

#### Priors

  

$${\mu }_{r}{\mathscr{\sim }}{\mathscr{N}}(\mathrm{0,1})(\mathrm{on}\,\mathrm{logit}\,\mathrm{scale},\,\mathrm{for}\,\mathrm{each}\,\mathrm{subregion}\,r)$$

  

$${\sigma }_{c}\sim \mathrm{Exponential}(1)$$

  

$$\phi \sim \mathrm{Exponential}()$$

The Normal (0,1) prior on the logit scale places approximately 95% of prior probability mass between prevalences of 0.1% and 99.9% and is therefore weakly informative — it regularises the estimates without meaningfully constraining the posterior away from the data.

#### National-level correction

Let ${\hat{p}}_{{Kenya}}^{{\rm{focal}}}$ denote the posterior predictive prevalence for Kenya derived from the focal study data, and let ${p}_{{Kenya}}^{{\rm{national}}}$ denote the observed national prevalence from the Kenyan national survey, computed as:

$${p}_{{Kenya}}^{{\rm{national}}}=\frac{\sum {k}^{{\rm{national}}}}{\sum {n}^{{\rm{national}}}}$$

The national correction factor δ* is then:

$$\delta =\frac{{p}_{{Kenya}}^{{\rm{national}}}}{{\hat{p}}_{{Kenya}}^{{\rm{focal}}}}$$

^*^δ is estimated from Kenya, and applied to all regions, representing an assumption of the model in the absence of additional national prevalence studies.

National-equivalent prevalence estimates for each subregion r are obtained by applying this factor to the subregion posterior:

$${\widetilde{p}}_{r}=\delta \cdot {p}_{r}$$

where ${p}_{r}$ is the marginal posterior prevalence for subregion r, integrating over the country-level random effects.

#### Posterior inference

Posterior distributions were estimated using MCMC sampling via the No-U-Turn Sampler (NUTS) as implemented in Stan (2.32.2), called through the brms (2.23.0) package in R (R version 4.5.1 (2025-06-13 ucrt). Four chains of 4000 iterations were run with 1000 warm-up iterations per chain, yielding 12,000 post-warmup draws. Convergence was assessed using the Gelman-Rubin diagnostic ($\hat{R} < 1.01$) and bulk ESS (ESS >400 for all parameters).

Using this approach, we generated regional prevalence ranges for four macro-regions, West and Central Africa, East and Southern Africa, South America, and Central America and the Caribbean (Supplementary Material 2).

To estimate the number of infections in the at-risk rural and urban populations we apply the following:

First, the at-risk rural population is determined:

$${A}_{c}\,=\,\left({P}_{c}{\rm{\cdot }}{R}_{c}\right)\,+\,\left({P}_{c}{\rm{\cdot }}{U}_{c}{\rm{\cdot }}{S}_{c}\right)$$

where *A*_*c*_ is the at-risk population of country (*c*), *P*_*c*_ is the total population, *R*_*c*_ the rural population, *U*_*c*_ the urban population, and *S*_*c*_ the slum dwelling population.

Then the infections are determined:

$${I}_{c}\,=\,{A}_{c}{\rm{\cdot }}{p}_{c}\,,\,{p}_{c}\, \sim \,{PERT}\left({\min }_{c}\,,\,{{mode}}_{c}\,,\,{\max }_{c}\right)$$

where *I*_*c*_ is the infections, *p*_*c*_ is the regional prevalence rate as sampled per country across a PERT distribution. The sampled *p*_*c*_ rate is applied equally to *A*_*c*_, representing a single rate for both rural and urban at-risk populations, considered as a combined at-risk population.

The inferred prevalence ranges are sampled as a probability distribution, followed by Monte Carlo simulation to account for the inherent uncertainty in this range. These expanded ranges, are, nonetheless, as assumption based on the use of a single national prevalence study from Kenya [22] set to derive the setting correction factor and a collection of highly heterogeneous prevalence estimates using a range of different methodologies, and thus, may not reflect the true prevalence of tungiasis, but rather serves as a preliminary estimate in the absence of concrete national prevalence data.

### Distribution of infections by age group

Tungiasis disproportionately affects children (individuals under 15 years of age) and those over 60, who are expected to experience the greatest disease burden. Based on the prevalences reported by age group in different randomized household surveys in Brazil, Nigeria, Uganda, Cameroon and Kenya [24, 41, 44–48], we attempted to model this by assigning an age distribution of cases for a given country as follows:

0–4 years old: 26% of total cases

5–15 years old: 34% of total cases

15–59 years old: 17% of total cases

60+ years old: 23% of total cases

We assume that these proportions are fixed across all countries for the sake of simplicity in the current model. However, to better account for the complexity of infection across different age groups, we assigned ranges for the proportion of each age group likely to experience moderate (> 6 embedded fleas) or severe (> 30 embedded fleas) tungiasis. These proportions, in turn, determine the application of comorbidity DWs, such as insomnia and/or missed school attendance. The proportion of moderate to severe cases were applied to the total number of infections within each for a given country, as follows.

0–4 years old: UNIF (0.33–0.55)

5–15 years old: UNIF (0.33–0.55)

15–59 years old: 0.3

60+ years old: UNIF (0.36–0.6)

Where UNIF denotes a uniform distribution, where all values across the range of probabilities (e.g. between 0.36–0.6) are equally likely to occur at the point of sampling the range. In the case of individuals aged 15–59 years, a single rate of 30% is applied to all individuals within this age group, as this age group is expected to experience the most severe effects of the disease at a lower rate than children and the elderly. However, age groups are used only for the estimation of infection rates within the population, and do not influence DALYs, as a single annual simulation model was used, to yield an estimate for the annual number of DALYs lost.

Finally, across all age groups, the total moderate-severe cases were subdivided, with 20% being classed as severe (> 30 embedded fleas), and the remaining 80% classed as moderate cases (> 6 but < 30 embedded fleas), with the appropriate DWs and co-morbidities applied accordingly. This represents a subdivision within the cases already classified as moderate-severe, herein defined as any individual with > 6 fleas embedded within the foot, and the 20% of moderate-severe cases classified as severe with comorbidities represents the individuals likely to be experiencing symptoms from > 30 embedded fleas. This split was determined based on prior studies which found 8%–15.1% of all individuals within a focal community study in Brazil [41] and Kenya [47] presented with > 30 embedded fleas. As the proportion of moderate-severe cases in our model varies by age group, and is represented by a range in high-risk age groups (33%–55% in 0–14 year olds; 36%–60% in 60+ year olds; Table 1), the 20% of moderate-severe cases classified as severe thus represents a conservative approximation where the number of cases selected as severe (> 30 embedded fleas) falls within the expected range (8%–15%) within the total population of infected individuals.

### Selection of countries

Selection of countries was based on a two-tiered co-author expert judgement. Initially, a preliminary list of countries was selected based on prior ecological niche modelling [15, 16, 26]. Regions within the zones identified as conducive to transmission of tungiasis is largely restricted to Sub-Saharan Africa, Central and South America. We therefore began by including all countries within West, Central, East, and Southern Africa and Latin America. From this list, we manually excluded all Caribbean islands, except Haiti, due to no reported or very limited transmission. We also excluded Chile, Argentina, Mexico, and Uruguay, as although these countries lie partially within regions known to be endemic, domestic cases are extremely rare, with limited reports of transmission, or highly localised transmission. Including them could have skewed the analysis due to their relatively large, combined population (> 50 million, the majority in Argentina). Additionally, we excluded the Maghreb and North Africa as a whole, and Gambia, due to limited evidence of transmission in these countries. A full list of excluded countries is provided in Supplementary Material 6, with associated DALY outputs and justification for exclusion.

### Input parameters

The model required a range of input parameters, summarised in Table 1. In addition, detailed population data, subdivided by age group, were necessary to calculate age-specific infection rates. These data were obtained in 5-year intervals from UN data available online (https://data.un.org/Data.aspx?d=POP&f=tableCode%3A22). The 5-year interval populations for each country included in the study were then summed and grouped in the following age categories: 0–4; 5–14; 14–59; 60+. The percentages of rural and urban populations were obtained from World Bank data available online (https://data.worldbank.org/indicator/SP.RUR.TOTL.ZS), and the percentage of urban populations residing in slums (https://data.worldbank.org/indicator/EN.POP.SLUM.UR.ZS). All data used in this study are based on the data available from 2024. Full population data can be found in Supplementary Material 2, and full background on input parameters can be found in Supplementary Material 3.

### Sensitivity analysis

A global probabilistic sensitivity analysis (PSA) was conducted to quantify the relative contribution of each model parameter to variance in the total global DALY estimate with fixed random seed 456. For each of 1000 iterations, all parameters were simultaneously sampled from their respective uncertainty distributions: prevalence from the regional PERT distributions derived from the Bayesian hierarchical model; DWs for mild, moderate, and severe tungiasis from PERT distributions informed by GBD reference values; severity fractions for the 0–14 and 60+ age groups from uniform distributions; the insomnia fraction from a uniform distribution; the moderate-to-severe case split from a uniform distribution centred on 0.20 with bounds 0.15–0.25; the seasonality amplitude from a uniform distribution spanning ±20% around the base value of 0.50; and age distribution proportions from a Dirichlet distribution centred on the baseline age structure with a concentration parameter of 100. The age distribution was treated as a compositional parameter ensuring each draw remained a valid set of proportions summing to one while allowing modest, correlated variation around the baseline. The comorbidity DW combination was treated as a single composite parameter, derived from correlated insomnia, mobility, and cognitive DWs via the GBD multiplicative combination rule. For each iteration, total global DALYs were computed by summing across all countries and all 52 weekly time steps. Standardised regression coefficients (SRCs) were then estimated by fitting a multiple linear regression of total global DALYs against the mean value of each parameter across that iteration, with each coefficient standardised by the ratio of the parameter standard deviation to the output standard deviation. The SRC² for each parameter approximates its proportional contribution to total output variance. Model linearity was confirmed by the coefficient of determination (R²) of the regression, with values exceeding 0.90 considered sufficient for reliable SRC estimation. Results are presented as a tornado plot ordered by absolute SRC, with direction of effect indicated. To determine the potential magnitude of the inclusion of the 17 countries within the ecological niche, but manually excluded, the model was run separately on these 17 countries (Supplementary Material 6), with all outputs collected for comparison to the included countries and totals.

## Results

### Global burden of disease

**Fig. 2:**
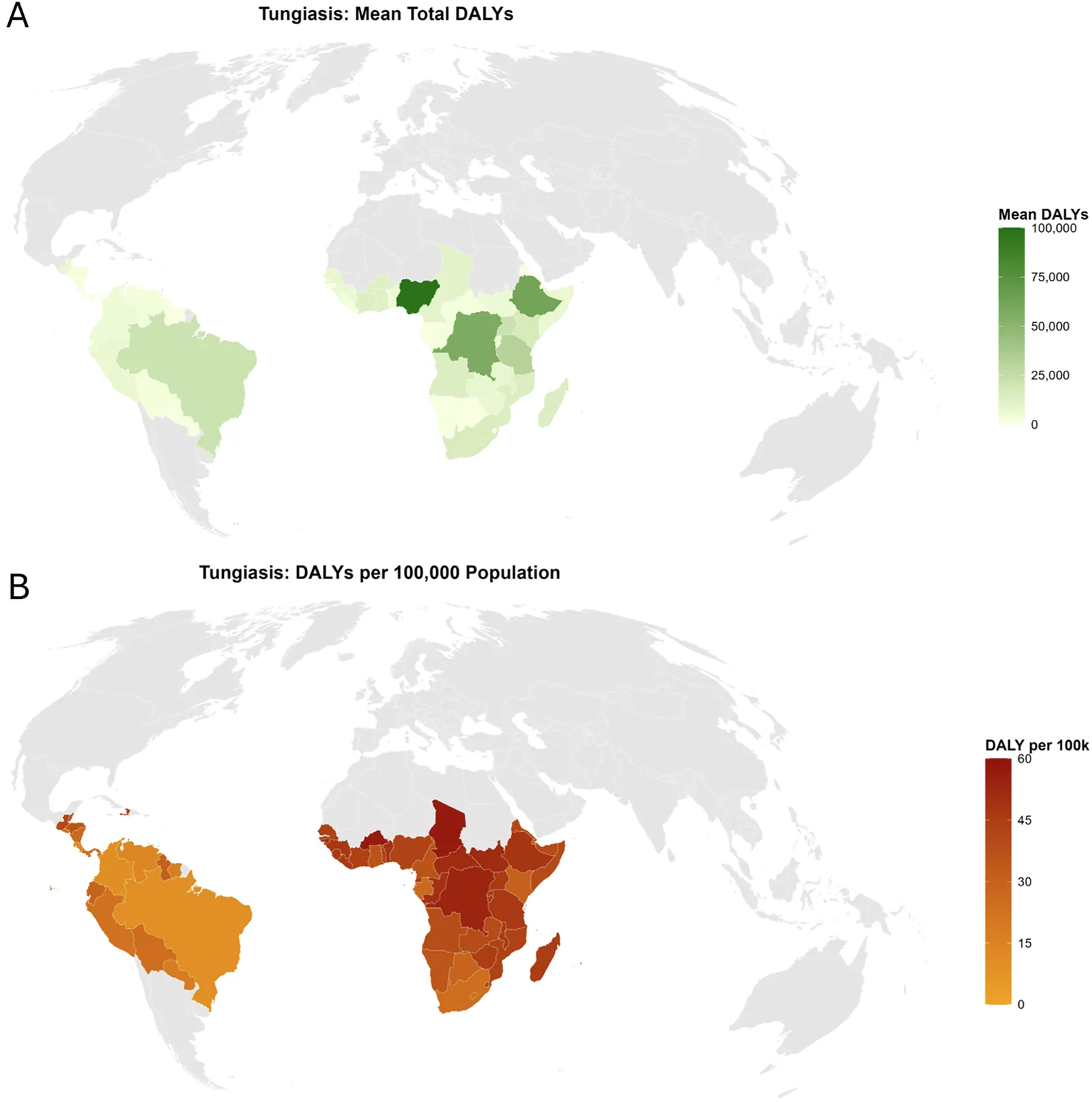
Global distribution of DALYs lost. **A** Mean total annual DALYs (green) across selected countries. **B** DALYs per 100,000 people (orange) across selected countries. Countries are shaded according to the amount of DALYs and DALYs per 100,000, with darker indicating greater, and lighter fewer, DALYs lost

We initially examined the distribution of DALYs lost across selected countries (Fig. 2; Supplementary Material 5). Across all countries included in the analysis, we estimate a total of 379,645 (95% uncertainty interval [UI]: 361,792–398,309) base DALYs lost annually, rising to 575,298 (95% UI: 548,948–602,597) when comorbidity DALYs are included. The countries with the greatest estimated burden were Nigeria (64,879 [61,716–68,427] base; 98,348 [93,396–103,535] with comorbidities), Ethiopia (41,006 [39,332–42,818] base; 62,116 [59,751–64,730] with comorbidities), the Democratic Republic of the Congo (37,505 [35,532–39,474] base; 56,835 [54,011–59,694] with comorbidities), Tanzania (20,565 [19,792–21,463] base; 31,166 [30,032–32,441] with comorbidities), and Uganda (14,762 [14,091–15,360] base; 22,367 [21,381–23,253] with comorbidities), which show the highest absolute numbers of DALYs lost. Overall, African countries account for nine of the ten countries with the highest burden, with Brazil (14,171 [13,426–14,971] base; 21,477 [20,339–22,700] with comorbidities) as the only non-African country in the top ten.

We then adjusted for population size and examined the estimated DALYs lost per 100,000 people. This was carried out to ensure that the proportional burden is accurately captured, as countries with large overall populations will have a higher absolute burden. At the global level, we estimate that 24.2 base DALYs, rising to 36.4 with comorbidities included, are lost annually per 100,000 people. We estimate that Burkina Faso (37.9 base; 57.5 [54.6–60.3] with comorbidities), Chad (37.8 base; 57.3 [54.3–60.3] with comorbidities), the Democratic Republic of the Congo (35.5 base; 53.7 [51.1–56.4] with comorbidities), South Sudan (34.3 base; 52.0 [49.9–54.0] with comorbidities), and the Central African Republic (34.2 base; 51.8 [49.1–54.4] with comorbidities) are expected to show the highest burden of disease per 100,000 people. Ethiopia (31.9 base; 48.3 [46.4–50.3] with comorbidities) and Uganda (30.3 base; 46.0 [43.9–47.8] with comorbidities) remain among the ten most affected countries; however, Nigeria (22.7 base; 34.4 [32.7–36.2] with comorbidities) and Brazil (6.7 base; 10.2 [9.6–10.8] with comorbidities) rank substantially lower, although these countries are amongst the most populous globally, proportionally, the disease burden is substantially lower. In the case of Brazil particularly, when adjusted for population size, it falls from the top ten most affected countries to the least affected, reflecting the higher rates of urbanisation, lower rates of slum dwelling population, and lower proportional burden relative to its population size.

### West and Central Africa

**Fig. 3:**
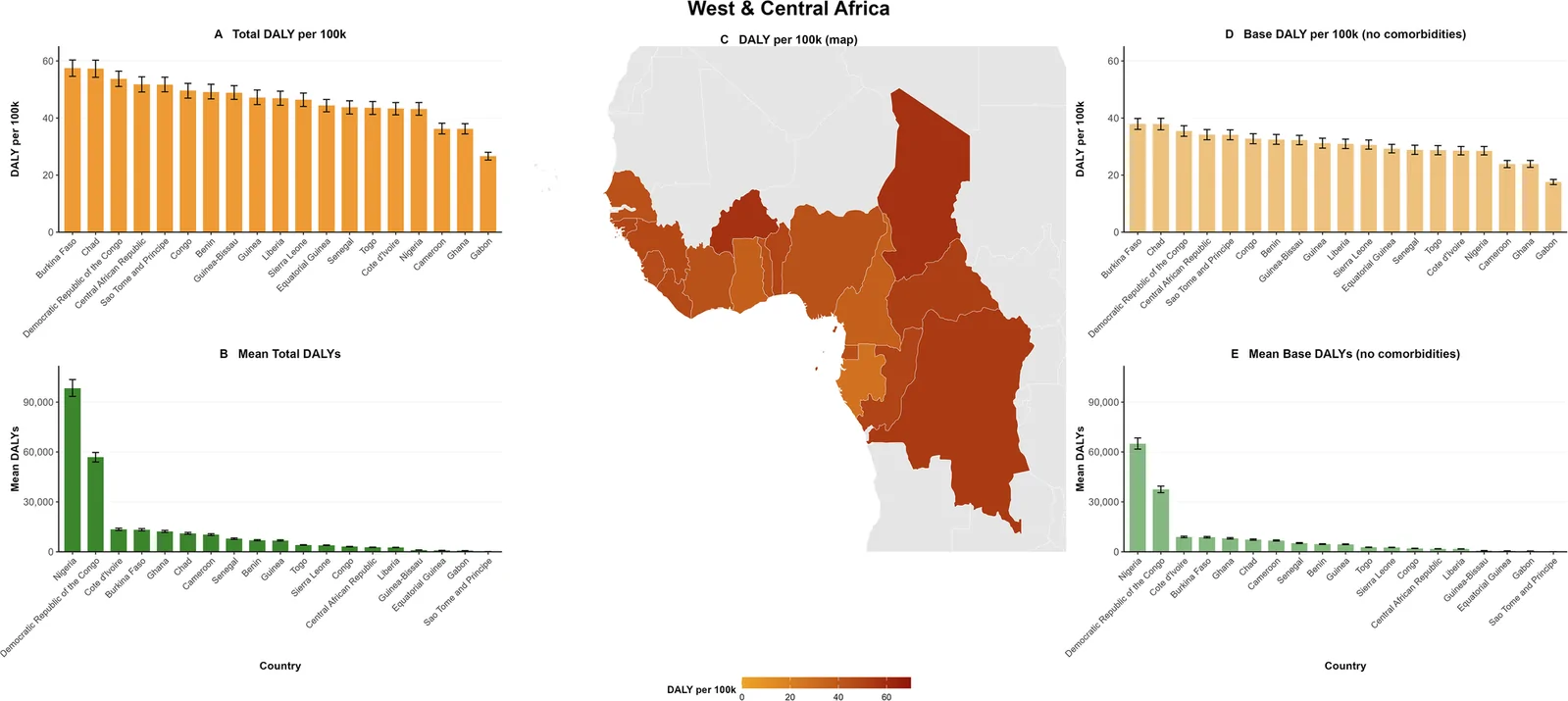
Regional distribution of DALYs per 100,000 people, and DALYs for West and Central Africa. **A** Mean DALYs lost per 100,000 people. **B** mean total DALYs lost. **C** Regional map shaded by mean DALYs lost per 100,000 people. **D** Base DALYs lost per 100,000 people (excluding comorbidities). **E** Mean base DALYs lost (excluding comorbidities). Error bars indicate 95% uncertainty intervals. Orange indicates mean DALYs per 100, 000 people, green indicates raw mean DALYs, after 1000 iterations of the 52-week simulation

Within West and Central Africa, we estimated that the greatest burden of disease occurs in Nigeria (64,879 [61,716–68,427] base; 98,348 [93,396–103,535] with comorbidities), the Democratic Republic of the Congo (37,505 [35,532–39,474] base; 56,835 [54,011–59,694] with comorbidities), Côte d’Ivoire (8915 [8441–9358] base; 13,510 [12,815–14,157] with comorbidities), Burkina Faso (8735 [8295–9177] base; 13,242 [12,576–13,895] with comorbidities), Ghana (8079 [7674–8493] base; 12,242 [11,631–12,848] with comorbidities), Chad (7310 [6931–7711] base; 11,071 [10,487–11,647] with comorbidities), Cameroon (6783 [6422–7128] base; 10,284 [9768–10,832] with comorbidities), Senegal (5218 [4929–5509] base; 7905 [7486–8324] with comorbidities), Benin (4575 [4345–4832] base; 6932 [6591–7314] with comorbidities), and Guinea (4488 [4244–4743] base; 6799 [6442–7176] with comorbidities) (Fig. 3).

When adjusted for population size, we estimated that the greatest burden of disease per 100,000 people within West and Central Africa occurs in Burkina Faso (37.9 base; 57.5 [54.6–60.3] with comorbidities), Chad (37.8 base; 57.3 [54.3–60.3] with comorbidities), the Democratic Republic of the Congo (35.5 base; 53.7 [51.1–56.4] with comorbidities), the Central African Republic (34.2 base; 51.8 [49.1–54.4] with comorbidities), Sao Tome and Principe (34.1 base; 51.7 [49.2–54.3] with comorbidities), the Republic of Congo (32.7 base; 49.6 [47.0–52.1] with comorbidities), Benin (32.4 base; 49.1 [46.7–51.8] with comorbidities), Guinea-Bissau (32.2 base; 48.9 [46.5–51.3] with comorbidities), Guinea (31.2 base; 47.2 [44.7–49.8] with comorbidities), and Liberia (31.0 base; 46.9 [44.5–49.4] with comorbidities) (Fig. 3).

### East and Southern Africa

**Fig. 4:**
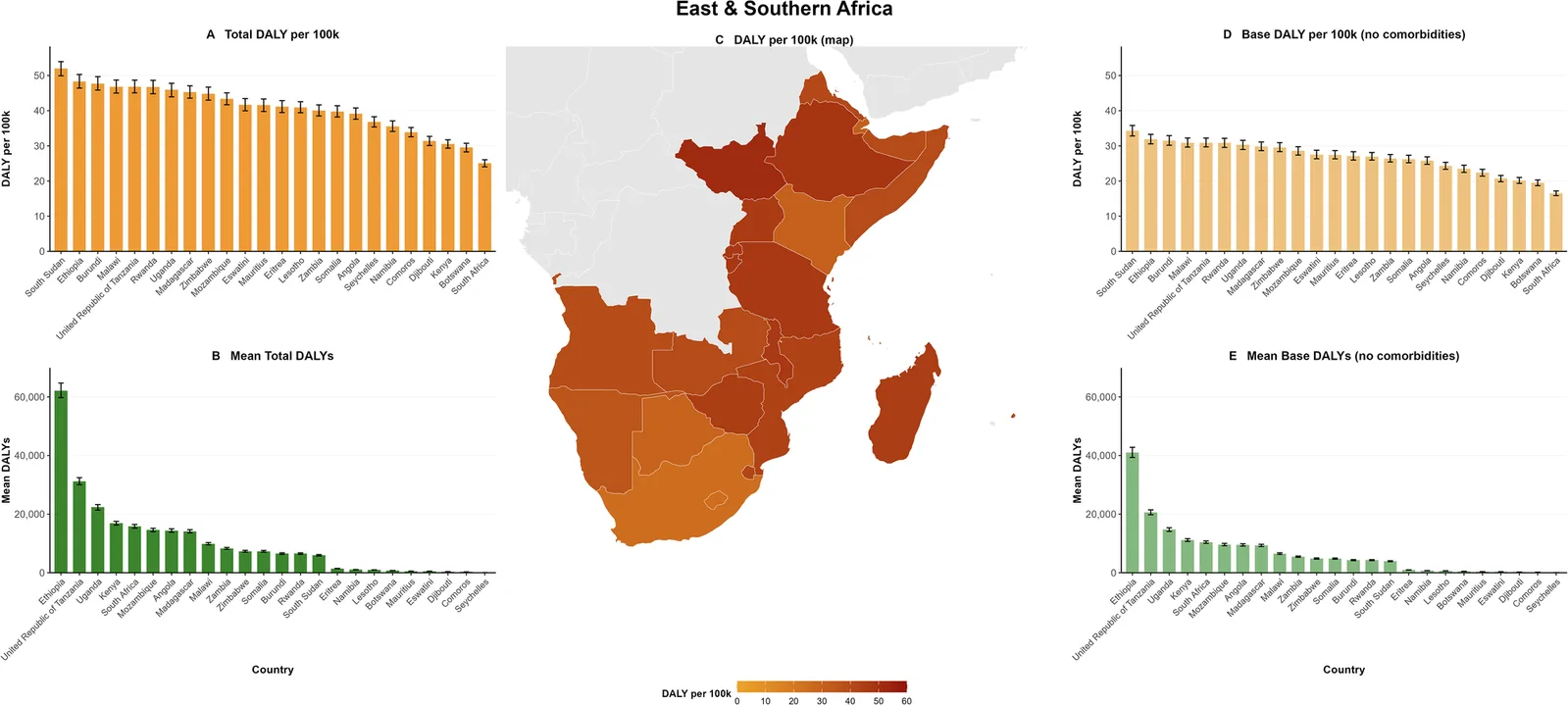
Regional distribution of DALYs per 100,000 people, and DALYs for East and Southern Africa. **A** Mean DALYs lost per 100,000 people. **B** Mean total DALYs lost. **C** Regional map shaded by mean DALYs lost per 100,000 people. **D** Base DALYs lost per 100,000 people (excluding comorbidities). **E** Mean base DALYs lost (excluding comorbidities). Error bars indicate 95% uncertainty intervals. Orange indicates mean DALYs per 100,000 people, green indicates raw mean DALYs, after 1000 iterations of the 52-week simulation

Within East and Southern Africa, we estimate that the greatest burden of disease occurs in Ethiopia (41,006 [39,332–42,818] base; 62,116 [59,751–64,730] with comorbidities), Tanzania (20,565 [19,792–21,463] base; 31,166 [30,032–32,441] with comorbidities), Uganda (14,762 [14,091–15,360] base; 22,367 [21,381–23,253] with comorbidities), Kenya (11,146 [10,690–11,625] base; 16,890 [16,238–17,555] with comorbidities), South Africa (10,440 [10,015–10,856] base; 15,815 [15,194–16,468] with comorbidities), Mozambique (9628 [9209–10,010] base; 14,593 [14,024–15,162] with comorbidities), Angola (9492 [9095–9877] base; 14,384 [13,804–14,982] with comorbidities), Madagascar (9316 [8935–9702] base; 14,121 [13,588–14,683] with comorbidities), Malawi (6519 [6255–6801] base; 9874 [9499–10,283] with comorbidities), and Zambia (5476 [5265–5705] base; 8295 [7980–8632] with comorbidities) (Fig. 4).

When adjusted for population size, we estimate that the greatest burden of disease per 100,000 people within East and Southern Africa occurs in South Sudan (34.3 base; 52.0 [49.9–54.0] with comorbidities), Ethiopia (31.9 base; 48.3 [46.4–50.3] with comorbidities), Burundi (31.5 base; 47.7 [45.9–49.7] with comorbidities), Malawi (30.9 base; 46.8 [45.0–48.7] with comorbidities), Tanzania (30.9 base; 46.8 [45.1–48.7] with comorbidities), Rwanda (30.9 base; 46.7 [44.8–48.6] with comorbidities), Uganda (30.3 base; 46.0 [43.9–47.8] with comorbidities), Madagascar (29.9 base; 45.3 [43.6–47.1] with comorbidities), Zimbabwe (29.6 base; 44.8 [43.0–46.7] with comorbidities), and Mozambique (28.6 base; 43.4 [41.7–45.1] with comorbidities) (Fig. 4).

### Western hemisphere

**Fig. 5:**
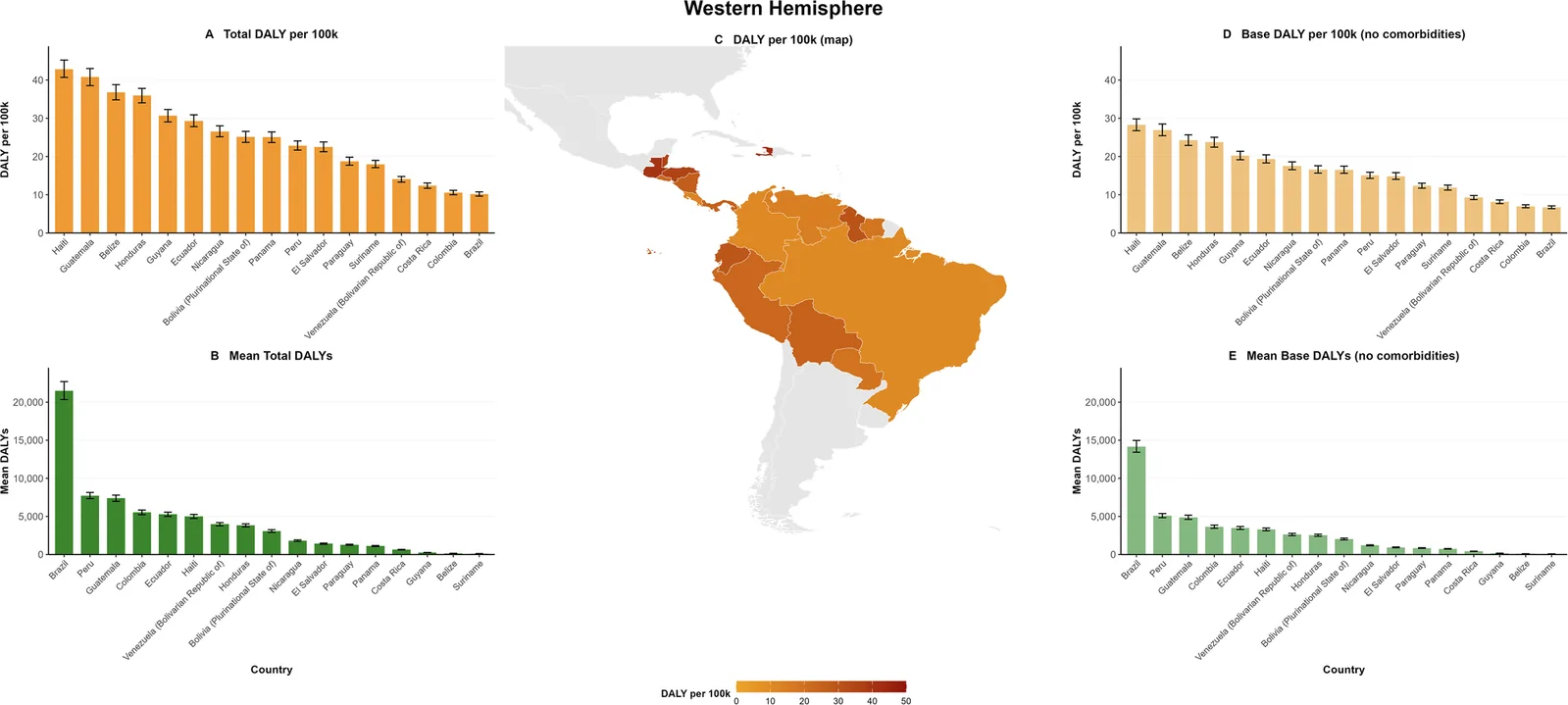
Regional distribution of DALYs per 100,000 population, and DALYs for Western Hemisphere endemic countries in the Caribbean, Central, and South America. **A** Mean DALYs lost per 100,000 people. **B** Mean total DALYs lost. **C** Regional map shaded by mean DALYs lost per 100,000 people. **D** Base DALYs lost per 100,000 people (excluding comorbidities). **E** Mean base DALYs lost (excluding comorbidities). Error bars indicate 95% uncertainty intervals. Orange indicates mean DALYs per 100k population, green indicates raw mean DALYs, after 1000 iterations of the 52-week simulation

Within South America, we estimate that the greatest burden of disease occurs in Brazil (14,171 [13,426–14,971] base; 21,477 [20,339–22,700] with comorbidities), Peru (5095 [4831–5379] base; 7720 [7336–8145] with comorbidities), Colombia (3645 [3450–3858] base; 5523 [5231–5835] with comorbidities), and Ecuador (3479 [3293–3673] base; 5270 [5000–5554] with comorbidities). When adjusted for population size, we estimate that the greatest burden of disease per 100,000 people occurs in Guyana (20.2 base; 30.7 [29.0–32.3] with comorbidities), Ecuador (19.3 base; 29.3 [27.8–30.9] with comorbidities), Bolivia (16.6 base; 25.1 [23.7–26.6] with comorbidities), and Peru (15.1 base; 22.8 [21.7–24.1] with comorbidities) (Fig. 5).

Within Central America and the Caribbean, we estimate that the greatest burden of disease occurs in Guatemala (4877 [4611–5164] base; 7391 [6983–7795] with comorbidities), Haiti (3289 [3113–3472] base; 4985 [4735–5261] with comorbidities), Honduras (2527 [2389–2664] base; 3829 [3622–4024] with comorbidities), Nicaragua (1195 [1129–1268] base; 1810 [1717–1912] with comorbidities), and El Salvador (937 [884–996] base; 1419 [1341–1502] with comorbidities). When adjusted for population size, we estimate that the greatest burden of disease per 100,000 people was in Haiti (28.3 base; 42.8 [40.7–45.2] with comorbidities), Guatemala (26.9 base; 40.8 [38.5–43.0] with comorbidities), Belize (24.2 base; 36.8 [34.8–38.8] with comorbidities), Honduras (23.7 base; 36.0 [34.0–37.8] with comorbidities), and Nicaragua (17.5 base; 26.5 [25.2–28.0] with comorbidities) (Fig. 5).

### Sensitivity analysis

**Fig. 6:**
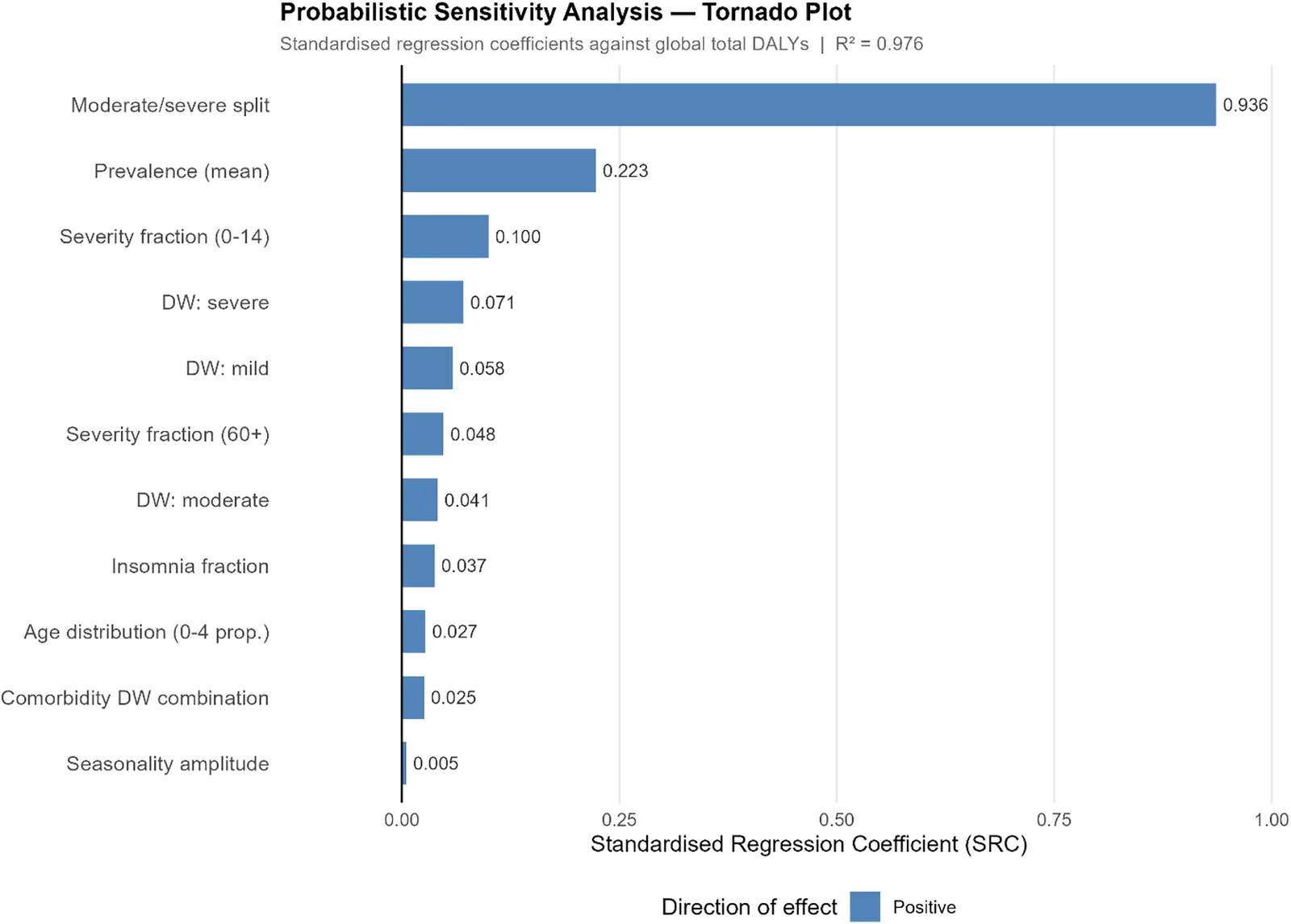
Probabilistic sensitivity analysis tornado plot showing standardised regression coefficients (SRCs) for all model parameters against global total DALYs across 1000 iterations. Parameters are ordered from top to bottom by decreasing variance contribution (SRC²)

We found the moderate/severe case split to be the dominant source of output variance (SRC = 0.88), followed by regional prevalence (SRC = 0.24) (Fig. 6). All remaining parameters individually contributed less than 15% of output variance. The high coefficient of determination (R² = 0.976) confirms sufficient model linearity for SRC-based sensitivity analysis. Positive SRCs indicate that increasing the parameter value increases total DALYs; the absence of negative bars indicates that all parameters exert a positive directional effect on the DALY estimate.

Finally, we examined the effect on the model for the 17 excluded countries (Supplementary Material 6). We found that inclusion of these countries would have increased the total DALYs by 32,103 (30,355-33,927), and 48,644 (46,085-51,331) with comorbidities included, representing an increase of an increase of 8.5% of the total DALYs reported.

## Discussion

In this study, we provide a preliminary estimate of the global burden of tungiasis, which to our knowledge represents the first quantitative assessment of this extremely neglected NTD. Our findings indicate that tungiasis imposes a substantial burden on global health, mostly centred on Sub-Saharan Africa, with an estimated 379,645 DALYs lost annually across endemic countries, rising to 575,298 when comorbidities are included. However, comorbidity estimations, particularly in East Africa, come with the caveat that there is potential overlap with podoconiosis, with between 10-20% of individuals affected by podoconiosis in certain communities were co-infected with tungiasis [25]. In Ethiopia, the most affected country globally, is estimated to lose 172,073 DALYs per annum based on 2017 estimates [49]. Our own estimate of DALYs lost due to tungiasis is lower at 62,116, although there may be some overlap in these values, particularly when considering comorbidity burden, it must be stated that these are distinct estimates, and the figures are not additive. This novel estimate, nonetheless, positions tungiasis well within the range of other NTDs in terms of global disease burden. The burden of tungiasis is comparable to that of *Ascaris lumbricoides* (754,000) [40, 50] with comorbidities but remains below that of the STHs as a whole (1.38 million) [33], and schistosomiasis (1.64–2.19 million) [34, 40, 51]. Scabies, a comparatively clinically similar ectoparasitic skin infection, is estimated to account for up to 5.3 million DALYs globally based on 2021 analyses [38]. Expectedly, our estimates for tungiasis are substantially lower, given its much more restricted and heterogenous geographic distribution [38, 52]. This is an important consideration in the context of the DALYs per 100,000 people. We estimated that, across the endemic countries included in this study, 24.2 base DALYs, rising to 36.4 with comorbidities included are lost per 100,000 people (based on the total population for countries included within our analysis). This figure is far higher than that most recently reported for STHs (18.84 [12.61 – 27.31]) [33], and schistosomiasis (21.895 [12.937 – 37.278]) [53], although it is of importance to note that the burden for both these infections has decreased substantially since the onset of global control programmes [33, 53], and are based on different methodologies (endemic-country vs global). Our estimate is almost identical to that of the burden of scabies in Sub-Saharan Africa (24.9 [13.8 – 40.2]), although lower than the overall global burden per 100,000 (68.7 [38.3 – 113.3], and those reported for East Asia (124.4 [69.7 – 205.5]) Andean (95.8 [53.2 – 154.9) and Central America (85.2 [47.3 – 140.0]) [38]. This, in part, may be attributable to an essentially more concentrated disease burden than that of STHs and schistosomiasis, which are both endemic to additional highly populated regions in Asia. Based on prior analyses, the total population living in areas ecologically conducive to *T. penetrans* transmission is 1.118 billion, concentrated exclusively in Central and South America [26], and Sub-Saharan Africa [16]. In contrast, an estimated 1.5 billion people are infected with STHs, and a total of 5.3 billion live in at risk areas [33]. We, thus, see a similar overall disease burden when compared to *Ascaris lumbricoides*, however, with a more limited geographic distribution. A further possible attributable factor relates to the disease aetiology itself. Tungiasis, as an ectoparasitic infection primarily affecting the feet, leading to secondary sequelae often related to removal practices, has the capacity to cause substantial pain, discomfort, and impairs mobility, sleep, and daily functioning [54]. This is in contrast to STHs and schistosomiasis, which tend to exert more long-term, chronic effects [55]. These differences further highlight an important strength of the DALY approach when considering disease burden assessment, and represent a measure of a loss of healthy life [40], which can be severely affected by ectoparasites such as *Tunga* spp. and scabies, without there being an immediate or significant danger to the individuals’ overall health, or risk of death. Indeed, the deleterious effects of discomfort and loss of sleep are often overlooked when considering disease burden [56, 57]. We have, therefore, sought to directly address this in our assessment of the potential burden of tungiasis, and have endeavoured to include appropriate DWs to take account of the complex and variable profile of symptoms which infected individuals may experience. It must be stated finally, however, that direct comparisons between helminth and ectoparasitic disease are difficult, due to their vastly different disease aetiologies and transmission dynamics. The comparison rather exists to highlight the substantial disease burden likely exerted by tungiasis, within the context of more high-profile NTDs.

It is also worth noting that STHs, for example, have experienced a substantial reduction in DALYs lost over the past several decades [33, 58], largely due to concerted control efforts and elimination programmes which reduced DALYs from 6.3 million to 3.5 million between 2000 and 2019 [59], and a further reduction to 1.38 million by 2024 [31]. This highlights the important comparison between a group of NTDs that have been the subject of extensive and highly successful control programmes, and one that is essentially still not targeted with interventions by most endemic countries. This may be due to the lack of evidence-based treatment and prevention options in endemic areas; lack of knowledge of the disease, its burden and distribution; competition for limited resources by other diseases considered more important and stigma and discrimination which is widespread among endemic populations [60, 61]. In this context, it is also essential to note the value of quantification of disease burden and the important role it plays in supporting the case for increased investment in research, and control measures [31, 32, 62], although this may not directly translate to a proportional increases in healthcare related funding in endemic countries in the global south [63]. While funding to DALY ratios, for example, in the Brazilian public healthcare system generally do not accurately proportionally reflect the actual DALY burden, with a tendency for skewed overinvestment relative to Zika and Malaria, and underinvestment relative to DALY burden for Chagas disease [63], DALYs are nonetheless important determinants of research agenda [31]. A reduction in DALYs is also seen as a key indicator for success in the control of an NTD [64]. Thus, NTDs that do not have a quantified burden of disease may struggle to attract funding in the same way as those that have been previously included within global burden of disease studies. However, there is also the risk of underestimating the global burden due to the sparse availability of local prevalence data, which can in some cases negatively affect the perception of the disease [65]. Taking this into account, we have sought to balance these concerns against the risk of overestimating the burden of tungiasis, so that this work can serve as an effective call to action for further research into this severely neglected disease.

We observed a high degree of variation in the estimated burden of disease at the regional level, with Sub-Saharan African countries being disproportionately affected. This pattern is expected and is directly related to the estimation of the at-risk population and the size of countries. It is worth noting that our estimates of the at-risk population (Supplementary Material 3) are comparable to those previously estimated for endemic areas [15, 16, 26]. For example, we estimate a total at-risk population of approximately 1.056 billion (Methods; Supplementary Material 3), whereas prior estimates were 668 million for Sub-Saharan Africa [16], and 450 million for the Americas [26], totalling 1.118 billion. This slight discrepancy largely reflects differences in how the at-risk population was defined, versus the population living in at-risk areas for transmission. Previous analyses relied on geostatistical and ecological niche modelling [16, 26] to predict the populations living within possible endemic areas. Our study used a more a sociodemographic approach, with World Bank data as a proxy for targeting populations likely to live in conditions that promote transmission, such as lack of sanitation or earthen flooring. As such, we considered the entire rural, and slum dwelling urban populations to be at-risk, although this may not fully reflect the actual at-risk population, as the presence of conducive conditions does not necessarily imply that transmission will take place. In light of this, we have opted for a mixed approach, where we have in general followed ecological niche modelling [16, 26], but have supplemented this with evidence of local transmission, or lack thereof. We made use of available data from the world bank on the population residing in slums or informal urban settlements likely to be conductive to transmission. It is, finally, worth mentioning that prior ecological niche modelling was used to estimate the total population living in at-risk areas, rather than the narrower at-risk population, modelled by rural and urban slum populations as a proxy herein. There are fundamental conceptual differences between these two estimates. Nonetheless, both estimates are based on assumptions, which may not accurately reflect conditions on the ground. However, in the absence of more detailed and large-scale national prevalence studies, this is the best available data, and as such, we have opted for the more conservative approach, with a narrower definition of the at-risk population, to mitigate the risk of overestimation of the burden of disease.

When considering individual country results, Ethiopia, Nigeria, and the Democratic Republic of Congo are estimated to experience a burden of disease between 50,000–100,000 DALYs lost annually. This is to be expected, as all three countries have populations greater than 100 million, and large rural and urban slum populations with the rural population comprising 77% of the total population, and 64% of the urban population in residing in slums in Ethiopia, 46% rural, and 48% of the urban population residing in slums in Nigeria, and 47% rural, and 78% of the urban population residing in slums in the Democratic Republic of Congo. The same pattern is observed when disease burden is expressed per 100,000 people, with the highest estimated burdens in Burundi, Malawi, and Rwanda, each with less than 20% of their population residing in urban areas, and a high proportion of urban residents living in slums or informal settlements (as per World Bank, 2024). In contrast, South and Central America and the Caribbean showed much lower overall disease burden both in absolute and relative terms, reflecting the assumption that higher rates of urbanisation and generally greater access to sanitation reduce the likelihood of transmission [66–68]. Nonetheless, this does represent a simplification, does not take account of the substantial variations possible in this provision and should be considered in future work. A notable exception to this pattern was Guyana, which exhibits a high overall burden when adjusted for population, reflecting its relatively low rate of urbanisation compared to the rest of South America (as per World Bank, 2024; https://data.worldbank.org/indicator/EN.POP.SLUM.UR.ZS). It is for this reason that we included additional parameters to attempt to balance this, including the use of a prevalence range, sampled across a probability distribution, and the inclusion of a Monte Carlo simulation in order to provide a range of different outcomes based on variable prevalence across 1000 iterations. Nonetheless, despite the inherent uncertainties in our approach, many of our findings are largely consistent with previous studies examining the contexts of local prevalences in Africa [16, 17, 22, 24, 26] and South America [26, 28].

Analysis of the tungiasis burden is complex, due to the wide range of symptoms and comorbidities which result from the infection. This complexity is further compounded by the lack of defined DWs for tungiasis, necessitating the use of approximations based on a range of symptoms potentially caused by the disease. To address this, we defined three distinct manifestations of the disease based on the number of embedded sand fleas [41]. We followed an approach recently used to quantify the global burden of toxocariasis [37], a similarly neglected disease not previously included in global burden of disease assessments. To account for comorbidities, we also followed this approach taken for toxocariasis [35], assigning DWs to capture morbidities such as impacts on education among school-age children, based on reported difficulties in concentration and school absenteeism [7–9]. Additionally, we incorporated the deleterious effects of insomnia due to severe itching and pain, as well as decreased mobility limitations in severe cases, assigning DWs that best captured these effects. As this is a preliminary analysis, we have aimed to balance capturing the severe effects of tungiasis without overestimating the potential burden. As such, we have further chosen to separate the base DALYs from those caused by comorbidities. The DWs assigned here were therefore chosen to represent conservative estimates, and future analysis incorporating bespoke DWs may result in different estimates of the total DALYs lost. Finally, we included a seasonal component into our analysis, based on reported increases in transmission during the dry versus the wet season [17, 29]. We did not model the full dynamics of tropical weather systems, including short and long wet seasons, due to their complexity, but instead applied an average seasonal variation across all tropical regions. Incorporating more detailed seasonal dynamics would be valuable for follow-on work, but was found based on sensitivity analysis to have a low overall effect on model outputs, and therefore, given the already existing complexity of the model, was not taken forward for inclusion at this time. Finally, tungiasis is a zoonosis [13, 24], and likely exerts an additional burden on animals. However, in this initial study, we did not consider this aspect of disease burden and chose to focus exclusively on the burden in humans. It would, nonetheless, be valuable to consider this in follow up work, particularly using the zoonotic DALY (zDALY) approach [69].

A further important contribution of this study is to identify additional data needs necessary to move from the current preliminary estimation to a more robust quantification of the global burden of disease for tungiasis. First and foremost, large scale national prevalence studies, comparable to that already available in Kenya [22] are needed. Given the scale of these studies (approximately 20,000 individuals sampled in Kenya), we would recommend these be targeted to three to five countries with a high disease burden, based on existing local prevalence estimates, and our own DALY estimates, and ensuring global distribution, namely: Ethiopia, Nigeria, Brazil, Burundi, and Madagascar, for example. This would capture representative national prevalence estimates from West Africa, Central Africa, East Africa, and South America, covering countries with large populations, and smaller populations but high local reported prevalences. In addition, given the complexities identified with assigning comorbidities, and a purely symptom matching approach to assigning DWs, a methodology which, although it has precedent in previous work [37], nonetheless, relies on substantial assumptions. Given the high preliminary estimated disease burden identified in our work, it would be beneficial to carry out a structured expert elicitation within the GBD framework to assign tungiasis specific DWs. Finally, although tungiasis itself is not fatal, secondary infections and comorbidities arising from the infection can be. This is not an aspect considered in the current study, due to the difficulty of parameterising this aspect of the disease. Thus, future focal studies in high prevalence areas focused on accurately determining the comorbidity associated death rate will be essential to fully quantify the total disease burden.

### Policy implications

The DALY quantification framework has been shown to be a critical tool to raise awareness, make proportionate investment in research and control and monitor success of control programmes. An example is the development and implementation of control programmes against STH, targeted at most affected areas and populations at risk [34]. These programmes are coordinated by international organisations (WHO) and facilitated by donations of free medicines by the pharmaceutical industry and by financial support from international organisations and NGOs. Our results, together with the fact that this disease is fully treatable at a low cost [1, 2, 4, 6], calls for new, coordinated action to raise awareness, raise funds and develop a global control agenda. Tungiasis is a One Health issue, involving humans, (domestic) animals and an environmental reservoir [11]. We recommend that this issue is coordinated by WHO with involvement of other relevant international organisations such as WOAH, UNEP and STAR IDAZ IRC. This aligns with the stated goals of the NTD 2021-2030 roadmap of an integrated approach to reduce the impact of skin NTDs (https://www.who.int/activities/promoting-the-integrated-approach-to-skin-related-neglected-tropical-diseases).

It is important to note that this study unequivocally represents a preliminary estimate. Throughout, we have endeavoured to make clear that this estimate relies on a range of assumptions and approximations to enable a global analysis. First and foremost, due to the lack of national prevalence estimates for all endemic countries except Kenya [22]. We, therefore, relied on an assumed prevalence range partially based on this national estimate, which was used as an anchor point, supplemented with highly heterogeneous local prevalence estimates from a range of focal studies, and based on Bayesian inference to develop credible macro-regional prevalence range estimates. It is, furthermore, critical to note that this national prevalence estimate used as the anchor point was based on school aged children 8-14 years old. We sought to balance this assumption by, first, employing an expanded credible range based on this estimate, and second, applying this prevalence range only to the population judged to be most at risk, namely the rural and slum dwelling population. We, furthermore, excluded a number of countries that lie within the ecological niche for *Tunga* spp. transmission, but have limited reports of domestic transmission. However, this nonetheless still represents an assumption regarding urban transmission, but attempts to account for prevalence in peri-urban areas and informal urban settlements where transmission is endemic, particularly in countries such as Brazil [44]. Our approach was deemed necessary because, although local estimates are available for certain countries, reliable estimates are often lacking for many others, which influenced the decision to conduct a generalised global analysis based on Bayesian inference. This decision was taken because this study is intended to serve as a call to action for further research and funding to tackle tungiasis. Uncertainty remains due to the paucity of reliable national-level prevalence data for most endemic countries, and the necessity of assigning DWs based on symptom profiles, rather than disease-specific DWs. Particularly within East Africa, there is also potential overlap with comorbidity burden with podoconiosis [25, 49], which has not been specifically accounted for in the current study, as additional detailed data would be required to effectively quantify this. A further limitation in our current model is that we do not account directly for transmission, rather, we apply a prevalence range to the population as whole, albeit stratified by age. The development of compartmental transmission models, which directly account for environmental and zoonotic transmission, would address this. However, this approach was judged to be beyond the scope of the current study due to the substantial work necessary to properly parameterise such a model.

## Conclusions

In this study we provide a preliminary estimate of the global burden of disease attributable to human tungiasis. The estimated annual DALYs lost are comparable to that of *Ascaris lumbricoides*, and exceeded those reported for several other NTDs, including fascioliasis, toxocariasis, and cystic echinococcosis. Notably, the estimated DALYs lost per 100,000 people are higher than those of both STHs, and schistosomiasis. While this in a large part relates to the more concentrated severe disease burden of tungiasis, it nonetheless, also underscores the urgent need for strengthened intensive implementation of control measures and programs targeting tungiasis, particularly as it is easily treatable. These estimates should be interpreted as indicative rather than definitive. Nonetheless, this analysis represents an important step towards quantifying the health impact of tungiasis. The burden estimates reported here highlight the need for increased investment in surveillance, research and targeted control interventions for tungiasis.

## Supplementary information


Supplementary material 1: All model iterations: full output of the DALY model across all iterations per country
Supplementary material 2: GBD DWs from the 2019 global burden of disease study
Supplementary material 3: Table S1: Focal and national prevalence estimates used in the Bayesian hierarchical model; Figure S1: Forest plot showing ranges of focal prevalence estimates by country; Figure S2: Density plot of focal study prevalence distributions by region
Supplementary material 4: Bayesian hierarchical model derived regional prevalence estimates
Supplementary material 5: Final model outputs
Supplementary material 6: Model outputs for excluded countries with justification for the country’s exclusion
Supplementary material 7: Full model input data by country


## Data Availability

All model outputs are included within Supplementary Material. Model code is available at https://github.com/AlistairAntono/Tungiasis_DALY [70] with random seed available within the supplied code. All model inputs are available from public sources as detailed in the manuscript. Full model population data inputs are available in Supplementary Material 7.
