# Supplementary material for: Estimation of the global burden of tungiasis in endemic countries using a disability adjusted life-years modelling approach
Source: BMC Glob Public Health. 2026 Aug 31;4:85. doi: 10.1186/s44263-026-00318-2 (PMC13527991; doi:10.1186/s44263-026-00318-2)
Supplement: Supplementary file 1 [file 44263_2026_318_MOESM3_ESM.docx]

Prevalence summary

Table S1: Summary of prevalences from literature

| **Author** | **Year** | **Country** | **Macroregion** | **Region** | **Study design** | **Study settings** | **Sampling method** | **Sample size** | **Cases** | **Prevalence** | **Type** |
| --- | --- | --- | --- | --- | --- | --- | --- | --- | --- | --- | --- |
| Ugbomoiko et al. | [2007 [17]](https://pmc.ncbi.nlm.nih.gov/articles/PMC12604154/table/Tab1/#CR17) | Nigeria | SSA | WCA | CS | Community-based | Systematic random | 557 | 252 | 45.2 | Focal |
| Collins et al. | [2009 [18]](https://pmc.ncbi.nlm.nih.gov/articles/PMC12604154/table/Tab1/#CR18) | Cameroon | SSA | WCA | CS | Community-based | Simple random | 1151 | 610 | 53 | Focal |
| Mazigo et al. | [2012 [19]](https://pmc.ncbi.nlm.nih.gov/articles/PMC12604154/table/Tab1/#CR19) | Tanzania | SSA | ESA | CS | Community-based | Simple random | 586 | 249 | 42.5 | Focal |
| Njau et al. | [2012 [20]](https://pmc.ncbi.nlm.nih.gov/articles/PMC12604154/table/Tab1/#CR20) | Kenya | SSA | ESA | CS | Community-based | Simple random | 385 | 218 | 57 | Focal |
| Otta | [2015 [21]](https://pmc.ncbi.nlm.nih.gov/articles/PMC12604154/table/Tab1/#CR21) | Ethiopia | SSA | ESA | CS | Community-based | Simple random | 272 | 120 | 44.5 | Focal |
| Mutebi et al. | [2015 [22]](https://pmc.ncbi.nlm.nih.gov/articles/PMC12604154/table/Tab1/#CR22) | Uganda | SSA | ESA | CS | Community-based | Purposive | 236 | 80 | 33.9 | Focal |
| Ngunjiri et al. | [2015 [24]](https://pmc.ncbi.nlm.nih.gov/articles/PMC12604154/table/Tab1/#CR24) | Kenya | SSA | ESA | CS | Community-based | Simple random | 347 | 153 | 44 | Focal |
| Mwangi et al. | [2015 [25]](https://pmc.ncbi.nlm.nih.gov/articles/PMC12604154/table/Tab1/#CR25) | Kenya | SSA | ESA | CS | School-based | Simple random | 508 | 97 | 19.1 | Focal |
| Kampamba | [2015 [26]](https://pmc.ncbi.nlm.nih.gov/articles/PMC12604154/table/Tab1/#CR26) | Zambia | SSA | ESA | CS | Community-based | Simple random | 384 | 52 | 13.5 | Focal |
| Waruguru et al. | [2015 [27]](https://pmc.ncbi.nlm.nih.gov/articles/PMC12604154/table/Tab1/#CR27) | Kenya | SSA | ESA | CS | Community-based | Simple random | 428 | 129 | 30.1 | Focal |
| Namuhani & Kiwanuka | [2016 [23]](https://pmc.ncbi.nlm.nih.gov/articles/PMC12604154/table/Tab1/#CR23) | Uganda | SSA | ESA | CS | Community-based | Systematic random | 296 | 74 | 25 | Focal |
| Ramaswamy | [2016 [28]](https://pmc.ncbi.nlm.nih.gov/articles/PMC12604154/table/Tab1/#CR28) | Ethiopia | SSA | ESA | CS | Community-based | Simple random | 2676 | 514 | 19.21 | Focal |
| Albert & Kiprotich | [2016 [29]](https://pmc.ncbi.nlm.nih.gov/articles/PMC12604154/table/Tab1/#CR29) | Kenya | SSA | ESA | CS | Community-based | Systematic random | 1557 | 441 | 28.3 | Focal |
| Ugbomoiko et al. | [2016 [30]](https://pmc.ncbi.nlm.nih.gov/articles/PMC12604154/table/Tab1/#CR30) | Nigeria | SSA | WCA | CS | School-based | Purposive | 545 | 133 | 24.4 | Focal |
| Wafula et al. | [2016 [31]](https://pmc.ncbi.nlm.nih.gov/articles/PMC12604154/table/Tab1/#CR31) | Uganda | SSA | ESA | CS | Community-based | Systematic random | 422 | 95 | 22.5 | Focal |
| Walker et al. | [2017 [32]](https://pmc.ncbi.nlm.nih.gov/articles/PMC12604154/table/Tab1/#CR32) | Ethiopia | SSA | ESA | CS | School-based | Systematic random | 343 | 119 | 34.7 | Focal |
| Ugbomoiko et al. | [2017 [33]](https://pmc.ncbi.nlm.nih.gov/articles/PMC12604154/table/Tab1/#CR33) | Nigeria | SSA | WCA | CS | Community-based | Purposive | 593 | 252 | 42.5 | Focal |
| Wiese et al. | [2017 [34]](https://pmc.ncbi.nlm.nih.gov/articles/PMC12604154/table/Tab1/#CR34) | Kenya | SSA | ESA | CS | Community-based | Systematic random | 1086 | 324 | 25 | Focal |
| Girma et al. | [2018 [35]](https://pmc.ncbi.nlm.nih.gov/articles/PMC12604154/table/Tab1/#CR35) | Ethiopia | SSA | ESA | CS | Community-based | Systematic random | 366 | 250 | 58.7 | Focal |
| Elson et al. | [2019 [52]](https://pmc.ncbi.nlm.nih.gov/articles/PMC12604154/table/Tab1/#CR68) | Kenya | SSA | ESA | ECC | School-based | Purposive | 1,829 | 870 | 48 | Focal |
| Nsanzimana et al. | [2019 [36]](https://pmc.ncbi.nlm.nih.gov/articles/PMC12604154/table/Tab1/#CR36) | Rwanda | SSA | ESA | CS | School-based | Systematic random | 384 | 87 | 23 | Focal |
| Nyangacha et al. | [2019 [37]](https://pmc.ncbi.nlm.nih.gov/articles/PMC12604154/table/Tab1/#CR37) | Kenya | SSA | ESA | CS | Community-based | Simple random | 437 | 94 | 21.5 | Focal |
| Aballa | [2019 [38]](https://pmc.ncbi.nlm.nih.gov/articles/PMC12604154/table/Tab1/#CR38) | Kenya | SSA | ESA | CS | Community-based | Simple random | 78 | 30 | 39.7 | Focal |
| Mwai et al. | [2020 [39]](https://pmc.ncbi.nlm.nih.gov/articles/PMC12604154/table/Tab1/#CR39) | Kenya | SSA | ESA | CS | School-based | Simple random | 385 | 120 | 31.1 | Focal |
| Amare & Lindtjorn | [2021 [40]](https://pmc.ncbi.nlm.nih.gov/articles/PMC12604154/table/Tab1/#CR40) | Ethiopia | SSA | ESA | CS | School-based | Simple random | 861 | 468 | 54.4 | Focal |
| Tamene | [2021 [41]](https://pmc.ncbi.nlm.nih.gov/articles/PMC12604154/table/Tab1/#CR41) | Ethiopia | SSA | ESA | CS | School-based | Simple random | 487 | 138 | 5.83 | Focal |
| Anyaele & Enwemiwe | [2021 [42]](https://pmc.ncbi.nlm.nih.gov/articles/PMC12604154/table/Tab1/#CR42) | Nigeria | SSA | WCA | CS | Community-based | Simple random | 705 | 252 | 35.8 | Focal |
| Razanakolona et al. | [2022 [51]](https://pmc.ncbi.nlm.nih.gov/articles/PMC12604154/table/Tab1/#CR51) | Madagascar | SSA | ESA | CS | Community-based | Simple random | 2328 | 320 | 13.7 | Focal |
| Jorga et al. | [2022 [43]](https://pmc.ncbi.nlm.nih.gov/articles/PMC12604154/table/Tab1/#CR43) | Ethiopia | SSA | ESA | CS | Community-based | Systematic random | 821 | 427 | 52 | Focal |
| Mba et al. | [2022 [44]](https://pmc.ncbi.nlm.nih.gov/articles/PMC12604154/table/Tab1/#CR44) | Gabon | SSA | WCA | CS | Community-based | Purposive | 637 | 242 | 37.99 | Focal |
| Adriko | [2022 [3]](https://pmc.ncbi.nlm.nih.gov/articles/PMC12604154/table/Tab1/#CR3) | Uganda | SSA | ESA | CS | Community-based | Simple random | 984 | 276 | 28 | Focal |
| Ahirirwe et al. | [2023 [45]](https://pmc.ncbi.nlm.nih.gov/articles/PMC12604154/table/Tab1/#CR45) | Uganda | SSA | ESA | UCC | Community-based | Purposive | 412 | 60 | 15 | Focal |
| Owino et al. | [2023 [46]](https://pmc.ncbi.nlm.nih.gov/articles/PMC12604154/table/Tab1/#CR46) | Kenya | SSA | ESA | CS | Community-based | Simple random | 381 | 253 | 65 | Focal |
| Mwai et al. | [2023 [47]](https://pmc.ncbi.nlm.nih.gov/articles/PMC12604154/table/Tab1/#CR47) | Kenya | SSA | ESA | CS | Community-based | Simple random | 528 | 328 | 62.1 | Focal |
| Thielecke et al. | [2023 [48]](https://pmc.ncbi.nlm.nih.gov/articles/PMC12604154/table/Tab1/#CR48) | Uganda | SSA | ESA | CS | Community-based | Purposive | 1329 | 811 | 61 | Focal |
| Mtunguja et al. | [2023 [49]](https://pmc.ncbi.nlm.nih.gov/articles/PMC12604154/table/Tab1/#CR49) | Tanzania | SSA | ESA | CS | School-based | Simple random | 401 | 85 | 21.2 | Focal |
| Mutebi et al. | [2023 [50]](https://pmc.ncbi.nlm.nih.gov/articles/PMC12604154/table/Tab1/#CR50) | Uganda | SSA | ESA | CS | Community-based | Simple random | 4035 | 2534 | 62.8 | Focal |
| Teixera Santana et al | 2023 | Brazil | LA | SA | CS | Community-based | Observational | 555 | 45 | 8.108108 | Focal |
| Oscherov et al | 2008 | Argentina | LA | SA | CS | Community-based | Observational | 182 | 124 | 68.1 | Focal |
| da Silva Damazio and da Silva | 2009 | Brazil | LA | SA | CS | Community-based | Observational | 917 | 15 | 1.6 | Focal |
| Muehlen et al | 2006 | Brazil | LA | SA | CS | Community-based | Analytical | 496 | 253 | 51 | Focal |
| Bonfim et al | 2010 | Brazil | LA | SA | CS | Community-based | Analytical | 310 | 72 | 23.2 | Focal |
| Malmonet et al | 2020 | French Guyana | LA | SA | CS | Community-based | Observational | 692 | 2 | 0.289017 | Focal |
| Louis et al | 2014 | Haiti | LA | CAC | CS | Community-based | Observational | 383 | 119 | 31.1 | Focal |
| Joseph et al | 2006 | Haiti | LA | CAC | CS | Community-based | Observational | 177 | 132 | 74.57627 | Focal |
| Elson et al. | 2023 | Kenya | SSA | ESA | CS | Community-based | Analytical | 10865 | 147 | 1.35 | National |
| Elson et al. | 2023 | Kenya | SSA | ESA | CS | Community-based | Analytical | 10600 | 94 | 0.89 | National |
| Heukelbach et al | 2002 | Brazil | LA | SA | CS | Community-based | Observational | 1184 | 398 | 33.6 | Focal |
| Wilcke et al | 2002 | Brazil | LA | SA | CS | Community-based | Observational | 1185 | 398 | 33.6 | Focal |
| Carvalho et al | 2003 | Brazil | LA | SA | CS | Community-based | Observational | 241 | 132 | 54.8 | Focal |
| Muehlen et al | 2003 | Brazil | LA | SA | CS | Community-based | Observational | 548 | 281 | 51.3 | Focal |
| Pilger et al | 2008 | Brazil | LA | SA | CS | Community-based | Observational | 490 | 191 | 39 | Focal |

Figure S1: Forest plot of prevalences range by country from studies used for prevalence estimation

Figure S2: Density plot of prevalence distributions by sub region from focal high transmission zone studies in Central America and Caribbean (CAC), East and Southern Africa (ESA), South America (SA), and West and Central Africa (WCA)
